# Preparations and characterizations of effervescent granules containing azithromycin solid dispersion for children and elder: Solubility enhancement, taste-masking, and digestive acidic protection

**DOI:** 10.1016/j.heliyon.2023.e16592

**Published:** 2023-05-26

**Authors:** Duyen Thi My Huynh, Huynh Thien Hai, Nguyen Minh Hau, Huynh Kim Lan, Truong Phu Vinh, Van De Tran, Duy Toan Pham

**Affiliations:** a Department of Pharmaceutical and Pharmaceutical Technology, Faculty of Pharmacy, Can Tho University of Medicine and Pharmacy, Can Tho, 900000, Viet Nam; b Faculty of Pharmacy, Can Tho University of Medicine and Pharmacy, Can Tho, 900000, Viet Nam; c Department of Health Organization and Management, Can Tho University of Medicine and Pharmacy, Can Tho, 900000, Viet Nam; d Department of Chemistry, College of Natural Sciences, Can Tho University, Can Tho, 900000, Viet Nam

**Keywords:** Azithromycin, Solid dispersion, Effervescent, Bitter, Solubility

## Abstract

Azithromycin, a macrolide antibiotics, is one of the frequently used drugs in the children and elder. However, due to these population difficulty in swallowing and inefficient absorption, and azithromycin inherent poor solubility, bitter taste, and instability in the stomach acidic condition, it is a challenge to reach high oral bioavailability of this drug. To overcome these issues, we developed and characterized the effervescent granules containing azithromycin solid dispersion. Firstly, the solid dispersion was prepared, employing both wet grinding and solvent evaporation methods, with different types/amounts of polymers. The optimal solid dispersion with β-cyclodextrin at a drug:polymer ratio of 1:2 (w/w), prepared by the solvent evaporation method, significantly enhanced the azithromycin solubility 4-fold compared to the free drug, improved its bitterness from “bitter” to “normal”, possessed intermolecular bonding between the drug and polymer, and transformed the azithromycin molecules from crystalline to amorphous state. Secondly, the effervescent granules incorporating the solid dispersion were formulated with varied excipients of sweeteners, gas-generators, pH modulators, and glidants/lubricants. The optimal formula satisfied all the properties stated in the Vietnamese Pharmacopoeia. In summary, the final effervescent granules product could be further investigated in in-vivo and in clinical settings to become a potential azithromycin delivery system with high bioavailability for the children and elder.

## 1 Introduction

Azithromycin, a macrolide antibiotics, is one of the most frequently used drugs for bacterial infection in the children and elder, by suppressing protein synthesis and reducing bacterial growth by attaching to the submolecular structure of the 50S ribosome [1,2]. Nevertheless, the oral bioavailability of conventional azithromycin dosage forms is generally insufficient (∼37%) [3], owing to the fact that the children and the elder populations possess difficulty in swallowing and inefficient absorption [4,5]. Moreover, azithromycin has several inherent limitations, including poor aqueous solubility (∼0.1 mg/mL), bitter taste, and high degradation in the acidic pH of the stomach's internal environment [[6], [7], [8]]. To this end, the effervescent granules incorporating azithromycin solid dispersion demonstrate much potential for solving the aforementioned problems.

To achieve the pharmacological response, a drug solubility and dissolution rate are two of the factors determining the extent, release rate, and oral medication absorption [[9], [10], [11], [12], [13]]. Thus, it is vital to use efficient preparation processes to increase the drug solubility, consequently enhances its bioavailability. For this, the solid dispersion method is a useful, economical, and environmentally beneficial choice based on its combination with hydrophilic polymers. A solid dispersion system is defined as a solid-phase system in which one or more drugs are dispersed in one or more pharmacologically inert carriers [14,15]. Solid dispersion has been proven to significantly improve the solubility of poor soluble drugs such as azithromycin [16,17]. Regarding the solid dispersion fabrication, two commonly utilized methods are the wet grinding method and the solvent evaporation method [18], which possess the advantages of less organic solvents and drug decomposition avoidance [14,19], and the homogenization of the drugs and the polymers [14,20], respectively.

Several research papers have been published on the improving azithromycin physico-chemical properties by means of solid dispersion [18,[21], [22], [23], [24]]. For instance, S.C. Arora et al. (2010) prepared the azithromycin solid dispersion with an urea carrier using the solvent evaporation method, with a 2.11-times increase in the azithromycin solubility [22]. E. Adeli (2016) successfully prepared azithromycin solid dispersion with polyethylene glycol (PEG) with a release rate of 49.10% within 30 min [21]. Additionally, M. rong Zhao et al. (2016) stated that the azithromycin complexed with hydroxypropyl-cyclodextrin possesses a dissolution rate of 90.07% at 60 min [23]. The dispersion system of azithromycin:PEG 6000:sodium lauryl sulfate (1:4:2 w/w/w) improves the drug release to 99.56% at 60 min [25]. Lastly, J. Li et al. (2022) used Eudragit RL-PO as a polymer carrier and the system could enhance the dissolution rate of azithromycin to 88.37% at 60 min [18]. Nevertheless, to the best of our knowledge, no studies have considered the bitterness improvement of azithromycin solid dispersion, as well as evaluated the system ability to protect the drug from the gastric acidic condition.

To that end, this study developed and characterized the effervescent granules containing azithromycin solid dispersion to (1) enhance the azithromycin solubility, (2) improve the drug bitterness, and (3) counteract the effects of acidic pH in the stomach environment. Firstly, the azithromycin solubility at different pH was evaluated. Then, the solid dispersions, at different polymers and concentrations, were prepared by both the wet grinding and the solvent evaporation methods, and fully characterized. The optimal formula was then fabricated to be the effervescent granules, taken into account the types and amounts of numerous excipients. Finally, the product properties were determined based on the Vietnamese Pharmacopoeia.

## 2 Materials and methods

### 2.1 Materials

Azithromycin was supplied by DHG company, Vietnam (purity of 99%). PEG 6000, β-cyclodextrin (β-CD), and polyvinylpyrrolidone (PVP) K30 were bought from Sigma-Aldrich, Singapore. Saccharose, aspartame, aluminum hydroxide, magnesium hydroxide, aerosil, magnesium stearate, citric acid, sodium carbonate, sodium hydrocarbonate, calcium carbonate, sulfuric acid, hydrochloric acid, potassium dihydrophosphate, and phosphoric acid were imported from Xilong, China. Ethanol and distilled water were supplied by Hoa-Chat-Mien-Nam company, Vietnam. All other reagents, chemicals, and solvents were of pharmaceutical grades or higher.

The machines/equipment utilized in the study included ultrasonic machine (Wisd WUC-D22H, Korea), pH meter (Consort C1020, Belgium), double-beam UV–Vis spectrometer (Jasco V730, Japan), dissolution tester (Pharmatest, PTWS 120D-Germany), analytical balance (Ohaus-Germany), drying oven (Memmert-Germany), magnetic stirrers (IKA C-MAG HS10, Germany), infrared (IR) spectrometer (NICOLET 6700, Thermo), differential scanning calorimetry (DSC) machine (DSC 200 F3, Netzsch, Germany), and scanning electron microscopy (SEM) (S4800 Hitachi, Japan).

### 2.2 Evaluation of azithromycin solubility at different pH

The drug solubility at different pH of 1.2, 6.0, 6.8, and 7.0, simulating different parts of the gastrointestinal tract (i.e., the stomach, the duodenum, the small intestine, and the large intestine), was evaluated using the shaking method during a 120-min period [26]. For this, 37.5 mg of azithromycin was added to 500 mL of the respective media and the solution was shaked at 100 rpm at a temperature of 37 ± 0.5 °C. At each time interval of 5, 15, 30, 60, and 120 min, 10 mL of the sample was withdrawn, filtered, derivatized with H_2_SO_4_ 70% v/v (to perform the hydrolysis reaction, in which the drug glycoside bond was broken and the aglycone was exposed), and UV–Vis spectroscopic measured at 482 nm [27]. The azithromycin solubility was then calculated based on the standard curve (y = 0.0067x + 0.0554, R^2^ = 0.9999) and reported.

### 2.3 Preparation of azithromycin solid dispersions

Based on the literature [18,[21], [22], [23], [24]] and our preliminary investigations, 04 polymers were used, independently, for the fabrication of azithromycin solid dispersion, including the β-CD, PEG 6000, PVP K30, and Eudragit L100. Accordingly, for each polymer, three ratios of drug:polymer (w/w) were applied. These ratios were selected based on the previous reports and our preliminary results [28,29]. Additionally, two preparation methods were employed, namely the wet grinding method (N) and the solvent evaporation (DM) method. Thus, a total of 24 formulas were prepared (Table 1).

**Table 1:** Azithromycin solid dispersions formulation. N: wet grinding method, DM: solvent evaporation method, AZ: azithromycin, β*-*CD: β*-*cyclodextrin, PEG: polyethylene glycol, PVP: polyvinylpyrrolidone.

No.	Formula	AZ:polymer ratio (w/w)	No.	Formula	AZ:polymer ratio (w/w)
01	N_1	AZ:β*-*CD 1:1	13	N_3	AZ:PVP K30 1:4
02	N_1.1	AZ:β*-*CD 1:2	14	N_3.1	AZ:PVP K30 1:6
03	N_1.2	AZ:β*-*CD 1:3	15	N_3.2	AZ:PVP K30 1:8
04	DM_1	AZ:β*-*CD 1:1	16	DM_3	AZ:PVP K30 1:4
05	DM_1.1	AZ:β*-*CD 1:2	17	DM_3.1	AZ:PVP K30 1:6
06	DM_1.2	AZ:β*-*CD 1:3	18	DM_3.2	AZ:PVP K30 1:8
07	N_2	AZ:PEG 6000 1:4	19	N_4	AZ:Eudragit L100 1:4
08	N_2.1	AZ:PEG 6000 1:6	20	N_4.1	AZ:Eudragit L100 1:6
09	N_2.2	AZ:PEG 6000 1:8	21	N_4.2	AZ:Eudragit L100 1:8
10	DM_2	AZ:PEG 6000 1:4	22	DM_4	AZ:Eudragit L100 1:4
11	DM_2.1	AZ:PEG 6000 1:6	23	DM_4.1	AZ:Eudragit L100 1:6
12	DM_2.2	AZ:PEG 6000 1:8	24	DM_4.2	AZ:Eudragit L100 1:8

For the wet grinding method, azithromycin and polymers were weighed and mixed, followed by the addition of adequate ethanol as a wetting agent. The mixture was then ground thoroughly for 30 min to obtain the solid dispersion. Alternatively, for the solvent evaporation method, azithromycin and polymers were dissolved in the ethanol:water mixture (2:1 v/v) and stirred for 24 h. Then, the dispersions were heated to 65 °C to evaporate the solvents, stabilized in a desiccator for 24 h, ground, and extruded through a 0.2-mm sieve to obtain the solid dispersion.

### 2.4 Physicochemical evaluations of azithromycin solid dispersions

#### 2.4.1 Solubility

The process of measuring the azithromycin solubility in its solid dispersion form was conducted similarly to that of the azithromycin powder (section 2.2), at the optimal pH.

#### 2.4.2 Bitterness

The bitterness of azithromycin solid dispersions were evaluated in clinical settings with 06 healthy volunteers, aged 18–30, who had received training in bitterness and sweetness of food/cosmetics/pharmaceuticals formulations using the scoring method, by a physiological medical expert in Can Tho University of Medicine and Pharmacy hospital, following the British Pharmacopoeia guideline [24,26]. Ethically, the study was approved by the Can Tho University of Medicine and Pharmacy Ethics Council, No. 22.148.SV/PCT-HDDD, and all participants were informed of the research's aim, contents, rights, and obligations prior to participation. A series of test solutions with the following intensities were prepared, including (1) sucrose solution (0, 5, 10, 20, 40, and 60 g/L), (2) quinine hydrochloride standard bitter solution (0:100, 1:100, 1:80, 60:40, and 20:20 mL/mL), and (3) azithromycin solid dispersion solution (0, 0.5, 1, 1.5, and 2 g/L). The bitterness rating scale was set from low (score of 0) to high (score of 5). An intermediate score could be assigned with a difference of 0.5 points if the test solution's assessment of bitterness and sweetness falls between two neighboring standard solutions. All participants were required to test all solutions in a double-blind experiment, and rate the solution bitterness. The scores were then averaged and reported. To ensure the test reliability, the sucrose solutions should obtain scores of 0–1, and the standard bitter solutions should get scores of 4–5.

#### 2.4.3 Morphology, DSC, and IR analyses

The azithromycin solid dispersions were morphologically observed using the SEM. For this, 2–3 mg of the dispersion powders were sprayed onto a metal disc covered with conductive double-sided tape to create a slight layer of powder, followed by gold coating. SEM images of the particles surfaces were captured and observed in the vacuum chamber [[30], [31], [32]].

For the DSC measurements, on a DSC aluminum pan, 50–100 mg azithromycin solid dispersion powders were weighed and scattered to form a flat, solid layer of 2-mm height. Then, the lid, pre-punched with a tiny hole, was subjected to the pan. A reference pan was prepared similarly, without the solid dispersion. Finally, the two aluminum pans were put in the heating chamber, and analyzed in a temperature range of 30–250 °C at an increment of 5 ^°^C/min [[30], [31], [32]].

For the IR analysis, 1–2 mg of the samples (pure azithromycin powder, the pure polymers, and the azithromycin solid dispersion) were ground and mixed with 300–400 mg of KBr. The homogenized mixture was then compressed into 13-mm-diameter tablets with an 800-MPa pressing force. The IR analyses were performed with the received tablets, with a wavenumber range of 4000-400 cm^−1^.

### 2.5 Preparation of effervescent granules containing azithromycin solid dispersion

The effervescent granules containing azithromycin solid dispersion were prepared by simple mixing and grinding method. For this, all the excipients, except the solid dispersion, were ground and sieved through a 0.2-mm sieve, followed by gentle mixing with the solid dispersion. Then, the product was packed in suitable containers and kept in a desiccator until further uses. To find the optimal formula, various parameter were tested and the products were analyzed (Table 2). For this, the carbohydrate types (i.e., sucrose and aspartame) and amounts were first varied (formulas F1–F8), with the sweetness as the analyzed factor. Then, the optimal formula was varied the gas-generating excipients (formulas F9–F12), with the best formula being the one with a maximum effervescence onset time of <5 min, and a maximum effervescence duration of <8 min. Next, the pH modulators/buffers were investigated (formulas F13–F20). Since azithromycin is unstable at pH < 2, the pH modulator excipient is necessary to protect the released drug from degradation. For this, the in-vitro simulated gastric condition was employed, mimicking the stomach fluid in the fasting state (2 mL/min of gastric secretion [33] at a pH of 1–3 [34]). One effervescent granule dose was dispersed in 15 mL of distilled water, followed by the gradual addition of 50 mL of 0.1 N HCl until the pH reached values lower than 2. The solution pH was measured initially and after every HCl additions. The best pH modulator was the system that could tolerate the highest amount of HCl. Finally, the lubricant excipients were evaluated (formulas F21–F26), with the granule fluidity measured by the rest angle (angle of repose) following the Vietnamese Pharmacopoeia standard. The best formula should possess a rest angle of <40° for high flowability property.

**Table 2:** Effervescent granules containing azithromycin solid dispersion formulas. SD: solid dispersion that has a fixed ratio of azithromycin:β-CD of 1:2 w/w; x: the optimal carbohydrate content. All values have a unit of mg.

No.	SD	Aspartame	Sucrose	Na_2_CO_3_	NaHCO_3_	Citric acid	Na_2_HPO_4_	KH_2_PO_4_	Magnesium stearate	Aerosil
**F1**	300	100								
**F2**	300	120								
**F3**	300	140								
**F4**	300	160								
**F5**	300		1000							
**F6**	300		1200							
**F7**	300		1400							
**F8**	300		1600							
**F9**	300	x		400		400				
**F10**	300	x		800		400				
**F11**	300	x			800	400				
**F12**	300	x			400	400				
**F13**	300	120		400	800	400	200			
**F14**	300	120		400	800	400		200		
**F15**	300	120		450	800	400	250			
**F16**	300	120		450	800	400		250		
**F17**	300	120		475	800	400	275			
**F18**	300	120		475	800	400		275		
**F19**	300	120		475	800	400	275	300		
**F20**	300	120		475	800	400	275	400		
**F21**	300	120		475	800	400	275	400		10
**F22**	300	120		475	800	400	275	400		15
**F23**	300	120		475	800	400	275	400		20
**F24**	300	120		475	800	400	275	400	10	
**F25**	300	120		475	800	400	275	400	15	
**F26**	300	120		475	800	400	275	400	20	

### 2.6 Physicochemical evaluations of effervescent granules containing azithromycin solid dispersion

The physicochemical properties of the effervescent granules containing azithromycin solid dispersion were determined in terms of the appearance (by visual observation), drug qualitative and quantification (by chromatography, based on Vietnamese Pharmacopoeia), humidity (by drying method), flowability/rest angle (based on Vietnamese Pharmacopoeia), pH (by pH meter), mass uniformity (by weighing method), effervescence onset time and effervescence duration (by visual observation), and dissolution rate and percentage of drug release at 30 min (by dissolution tester, based on Vietnamese Pharmacopoeia). A summary of these experiments is presented in Table 3.

**Table 3:** Complete physicochemical properties determinations of effervescent granules containing azithromycin solid dispersion (n = 6).

No	Property	Testing method	Standard	Test outcome
01	Appearance	Visual observation	White dry homogeneous powder	Satisfied
02	Drug qualitative	Thin layer chromatography	Based on Vietnamese Pharmacopoeia	Satisfied
03	Drug quantification	High performance liquid chromatography (HPLC)	98–102%	Pass (99.68 ± 1.02%)
04	Humidity	Drying method (IR balance)	1.8 %-6.5%	Pass (4.7 ± 0.3%)
05	Flowability (Rest angle)	Based on Vietnamese Pharmacopoeia	<40°	Pass (33 ± 0.5°)
06	pH	pH meter	6–6.5	Pass (6.06 ± 0.05)
07	Mass uniformity	Weighing method	Based on Vietnamese Pharmacopoeia	Satisfied
08	Effervescence onset time	Observation (5 g granules/50 mL water)	<5 min	Pass (4.5 ± 0.2 min)
09	Effervescence duration	Observation (5 g granules/50 mL water)	<8 min	Pass (7.2 ± 0.3 min)
10	Dissolution rate	Dissolution tester, paddle, 100 rpm	Based on Vietnamese Pharmacopoeia	Satisfied
11	%Drug release at 30 min	Dissolution tester, paddle, 100 rpm	≥80%	Pass (88.1 ± 1.6%)

## 3 Results and discussions

### 3.1 Evaluation of azithromycin solubility at different pH

**Fig. 1:**
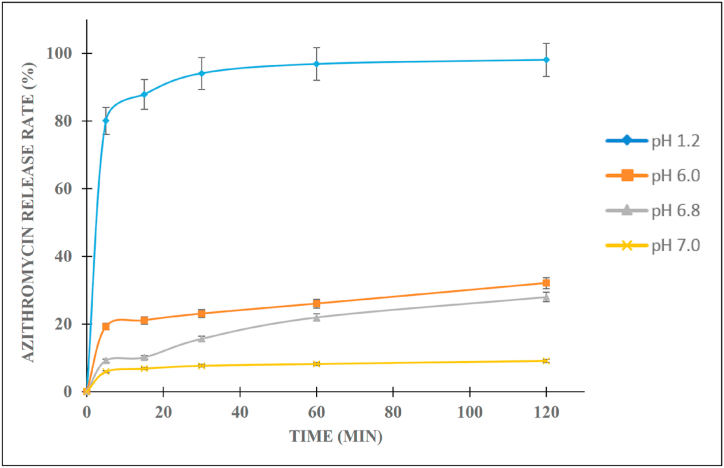
In-vitro release profiles of azithromycin in different pH solutions.

The solubility of azithromycin was determined sequentially in the solutions with pH 1.2, 6.0, 6.8, and 7.0. Since azithromycin is a weak base drug with its structure composing of two amines (CH_3_–N–) groups [35], the solution low pH protonates the molecules, consequently increasing the azithromycin solubility (Fig. 1). Thus, in pH 1.2, azithromycin dissolves up to 98.1%. However, it is decomposed in this condition due to the hydrolysis action of the ion H^+^ at the glycoside bonds [7]. On the other hand, at higher pH (i.e., pH 6.0), the azithromycin molecules mainly stay in the non-ionic form, hence, it could only dissolve as high as 21.12% at 120 min. Additionally, the azithromycin solubility at pH 6.8 and 7.0 were lower than that at pH 6.0, which could be due to the higher degradation of the drug in pH 6.8 and 7.0 [36]. Thus, pH 6.0 was utilized for the next experiment. Conclusively, at both low and high pH, the azithromycin oral bioavailability is considerably inadequate. To overcome this issue, azithromycin solid dispersions were utilized.

### 3.2 Preparations and physicochemical evaluations of azithromycin solid dispersions

#### 3.2.1 Solubility

**Fig. 2:**
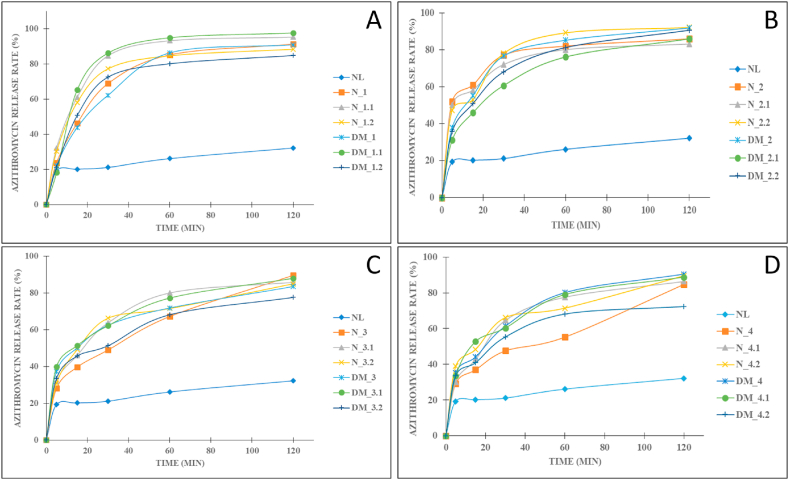
In-vitro release profiles of azithromycin solid dispersions prepared with the carrier of (A) β-cyclodextrin, (B) polyethylene glycol (PEG) 6000, (C) polyvinylpyrrolidone (PVP) K30, (D) Eudragit L100, in pH 6.0. NL: azithromycin powder; N: azithromycin solid dispersions prepared by the wet grinding method; DM: azithromycin solid dispersions prepared by the solvent evaporation method. The numbers (i.e., 1.1, 1.2) indicate the azithromycin and polymer ratios (Table 1).

The azithromycin solid dispersions with different types and amounts of polymers were formulated to select the optimal formulation (Table 1). All 04 investigated polymers of β-CD, PEG 6000, PVP K30, and Eudragit L100 could significantly improve the azithromycin solubility and dissolution profiles (Fig. 2(A-D)). Thus, for each polymer, one formula was selected for the bitterness tests.

Firstly, for the carrier β-CD, the polymer formed porosity on the surface of azithromycin, consequently protected azithromycin from crystallizing and accelerated the drug solubility and permeability. Similar results were reported, which demonstrated that the increment in solubility of the azithromycin solid dispersion with hydroxypropyl-cyclodextrin at a ratio of 10:1 (w/w) [23]. The formula DM_1.1 (azithromycin:β-CD 1:2 w/w ratio) yielded the highest increase in the drug solubility, with nearly 4 times enhancement. Hence, the formulation DM_1.1 was chosen.

Secondly, the azithromycin complex with PEG 6000, with the ratio of 1:6 w/w (formula N_2.2), increased the solubility of azithromycin to 78.01%, 3.69-times higher than that of the pure drug (21.12%) at 30 min. These data were in agreement with the previous work stating that PEG 6000 enhances the drug solubility to 2.7 times more than the pure drug [25]. Interestingly, an increase in the PEG 6000 amount (formula 2.3) decreases the azithromycin solubility, which could be because of the carrier high viscosity that can plummet the diffusion coefficient of azithromycin. In summary, formula N_2.2 was selected. Similar phenomenon was observed in the PVP K30 polymer, of which formula N_3 was chosen.

Finally, the solid dispersion with Eudragit L100 increased the drug solubility up to 80.16% at 60 min. However, when the ratio of EL100 increased, the solubility was not increased due to the viscosity of the polymer. These results were in agreement with the previous study [25]. Conclusively, the formulation DM_4 was selected.

#### 3.2.2 Bitterness

Four solid dispersion formulas, selected from the previous experiments, together with the pure azithromycin, were subjected to the bitterness tests in 06 healthy volunteers. The bitterness scores of the pure azithromycin powder, the DM_1.1, N_2.2, N_3, and DM_4 formulas were 3.05 ± 0.08, 0.81 ± 0.01, 3.2 ± 0.21, 1.23 ± 0.02, and 1.19 ± 0.01, respectively. Accordingly, the azithromycin solid dispersion with β-CD at a ratio of 1:2 (w/w), prepared by the solvent evaporation method (formula DM_1.1), could significantly reduce the azithromycin bitterness from “bitter” (score >3) to “normal” and “sweet” (score <1). This can be due to the fact that β-CD porous molecular structure successfully encapsulated the azithromycin molecules inside its empty cavities. Moreover, the mixture of azithromycin and β-CD formed additional hydrogen bonding (discussed in the next section), which better protected the solid dispersion particles from the bitter receptors on the human tongue. On the other hand, the solid dispersions with PEG 6000 and PVP K30 showed bitter tastes, possibly due to the high azithromycin release at 5 min (Fig. 2B and C). Interestingly, although the solid dispersion prepared with the polymer Eudragit L100 possessed a high drug release at 5 min, it had a low bitter score, which could be attributed to the hydrogen bonds formations [18]. Conclusively, the formula DM_1.1 was selected for further characterizations and for effervescent granules preparation.

#### 3.2.3 Morphology, DSC, and IR analyses

To characterize the azithromycin solid dispersion and to elucidate the interactions between azithromycin and β-CD, IR, SEM, and DSC analytical techniques were employed.

**Fig. 3:**
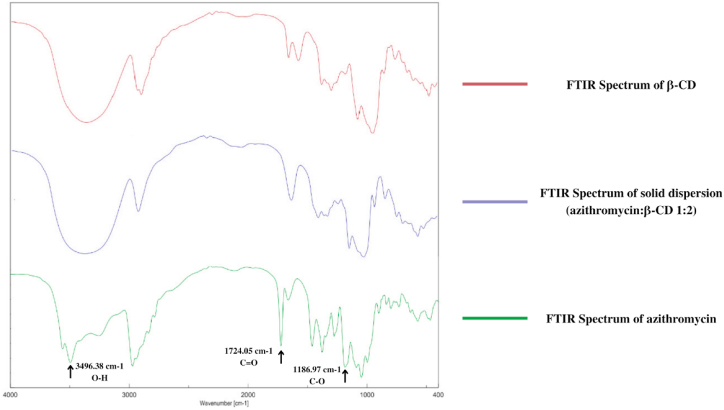
Infrared (IR) spectra of the pure azithromycin, the polymer β-cyclodextrin (β-CD), and the azithromycin solid dispersion with β-cyclodextrin at a ratio of 1:2 w/w.

Firstly, the IR spectra (Fig. 3) show that the pure azithromycin had a hydroxyl group (O–H), a carbonyl group (C=O), and a C–O group peaked at 3494.38 cm^−1^, 1724 cm^−1^, and 1186 cm^−1^, respectively. In the solid dispersion spectrum, the sharp peak of azithromycin O–H groups at 3494.38 cm^−1^ was replaced by an obtuse peak of β-CD hydroxyl groups, indicating that the drug azithromycin was successfully incorporated into the β-CD polymer network. Interestingly, in the azithromycin solid dispersion, the carbonyl peaks at 1724 cm^−1^ disappeared, suggesting the formations of intermolecular hydrogen bonding between the azithromycin carbonyl groups and the β-CD hydroxyl groups.

**Fig. 4:**
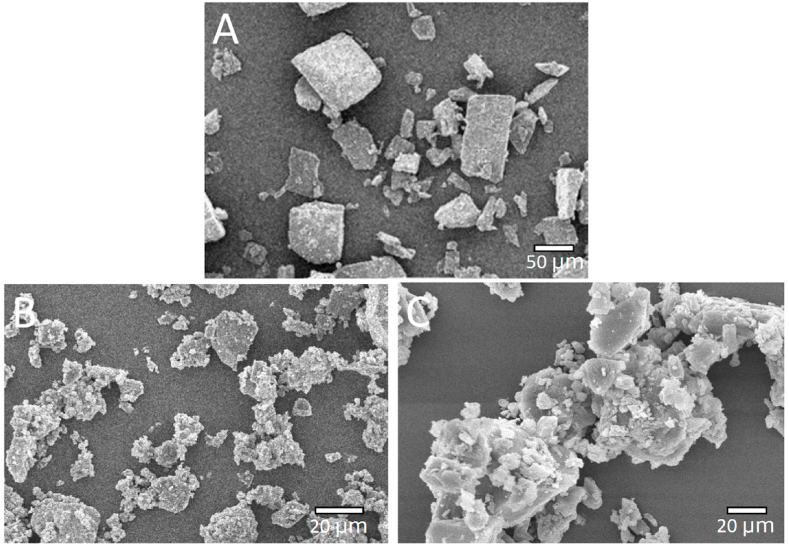
Scanning electron microscopy (SEM) images of the pure azithromycin (A, scale bar: 50 μm), the polymer β-cyclodextrin (B, scale bar: 20 μm), and the azithromycin solid dispersion with β-cyclodextrin at a ratio of 1:2 w/w (C, scale bar: 20 μm).

Secondly, SEM analysis revealed that the azithromycin powder possessed cubic-shaped crystals with sharp edges, constituting to its low solubility (Fig. 4A). On the other hand, the surfaces of the azithromycin solid dispersions were similar to the surface of β-CD, indicating that the β-CD particles had covered azithromycin surface and formed a complex between them (Fig. 4B and C). The new complex contained the formation of pores on the surface of azithromycin, which enhanced the drug solubility and accelerated drug dissolution.

**Fig. 5:**
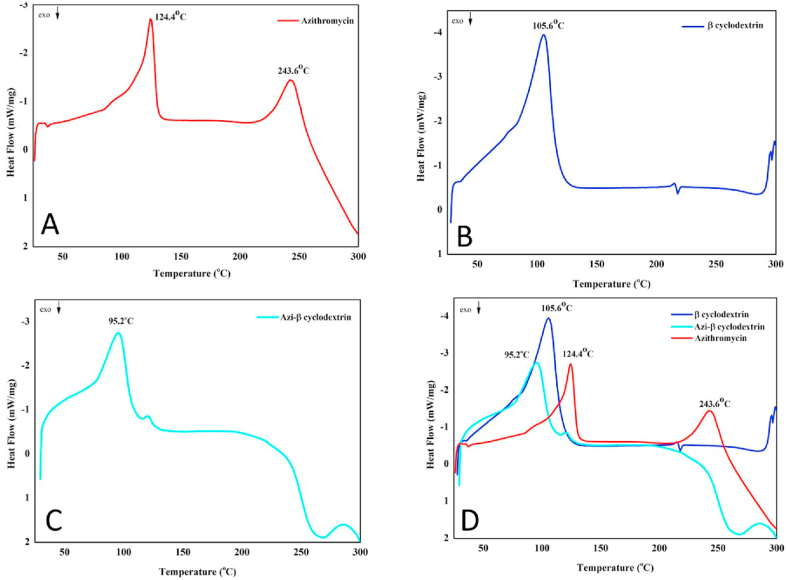
Differential scanning calorimetry (DSC) graphs of (A) the pure azithromycin, (B) the polymer β-cyclodextrin, (C) the azithromycin solid dispersion with β-cyclodextrin (azi-β cyclodextrin) at a ratio of 1:2 w/w, and (D) merged samples.

The high dissolution rate of azithromycin in the solid dispersion was also resulted from the drug amorphous state. By using DSC analysis, the amorphous state of azithromycin was confirmed (Fig. 5A). The DSC graph of the polymer β-CD showed an endothermic peak at 105.6 °C, indicating its melting point (Fig. 5B). Azithromycin powder had two endothermic peaks at 124.4 °C and 243.6 °C, corresponding to its crystalline melting point and degradation point, respectively [37]. Interestingly, the melting endothermic peak (124.4 °C) disappeared in the azithromycin solid dispersion, suggesting the drug azithromycin has been changed from crystalline to amorphous state (Fig. 5C and D).

### 3.3 Preparations and physicochemical evaluations of effervescent granules containing azithromycin solid dispersion

**Fig. 6:**
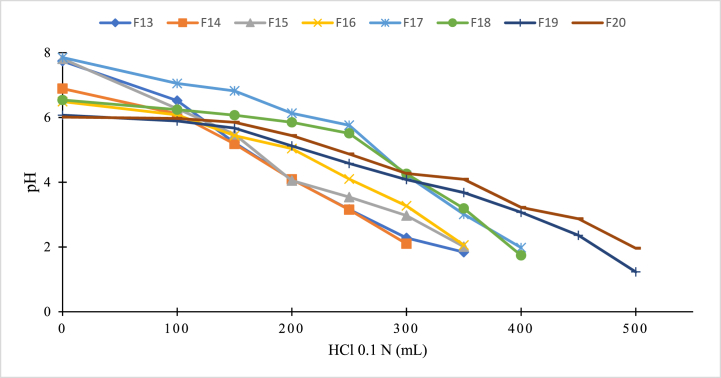
Comparison of the acidic neutralization ability of the effervescent granules containing azithromycin solid dispersion, with different pH modulators (formula F13–F20, Table 2), in the in-vitro simulated stomach condition.

The best solid dispersion formula (DM_1.1) was utilized to formulate the effervescent granules. To this end, various parameters of excipients were tested to select the optimal formulation (Table 2). Firstly, the best carbohydrates excipient was aspartam, with the quantity of 120 mg per unit (formula F2), due to its light sweet and non-moisturizer effect, as compared with the sucrose. Secondly, the NaHCO_3_ and citric acid were chosen as main gas-generating excipients (formula F11) since they produced the maximum effervescence onset time of <5 min (4.1 min), a maximum effervescence duration of <8 min (6.8 min), and a suitable pH of the solution after effervescing (5.83). Thirdly, the glycoside bonds of azithromycin with cladinose are not stable in the low pH (pH ≤ 2) due to the attacking of ion H^+^ (T_10%_ = 56.3 min), and when pH > 3, the azithromycin decomposition rate descreases 10 times [24]. Therefore, the pH modulators are necessary to maintain the system pH and to avoid drug degradation. For this, the pH modulators system of Na_2_CO_3_:Na_2_HPO_4_:KH_2_PO_4_ (475:275:400 w/w/w) (formula F20) was chosen since this system could maintain the pH > 2 at the largest HCl amount, provided suitable pH of the solution, and did not affect the effervescent ability (Fig. 6). Finally, aerosil (20 mg, formula F23) was selected as glidant in the formulation since it can improve the powder flowability (rest angle of <33 ± 0.5°). Conclusively, formula F23 was the best preparation of the effervescent granules containing azithromycin solid dispersion.

Finally, the complete physicochemical properties of the effervescent granules product was evaluated (Table 3). All properties were acceptable, according to the Vietnamese Pharmacopoeia.

## 4 Conclusions

This present work successfully developed and fully characterized the effervescent granules containing azithromycin solid dispersion for special subjects such as children and the elder, with the purposes of solubility and bitterness enhancement, along with stomach acidic protection. The optimal solid dispersion with β-cyclodextrin at a drug:polymer ratio of 1:2 (w/w), prepared by the solvent evaporation method, significantly increased the azithromycin solubility to nearly 4 times and improved its bitterness from “bitter” to “normal”. Analytically, the solid dispersion possessed intermolecular bonding between the drug and polymer, in which the drug was transformed from crystalline to amorphous state. Finally, the effervescent granules incorporating the solid dispersion, with optimal excipients types/amounts, satisfied all the properties stated in the Vietnamese Pharmacopoeia, indicating that the final product is suitable to be further researched in in-vivo and in clinical settings to become a dosage form containing azithromycin with high bioavailability that is appropriate for the children and elder.

## Author contribution statement

Duyen Thi My Huynh: Huynh Thien Hai: Conceived and designed the experiments; Performed the experiments; Analyzed and interpreted the data; Wrote the paper.

Nguyen Minh Hau: Huynh Kim Lan: Truong Phu Vinh: Van De Tran: Performed the experiments.

Duy Toan Pham: Conceived and designed the experiments; Analyzed and interpreted the data; Contributed reagents, materials, analysis tools or data; Wrote the paper.

## Data availability statement

Data will be made available on request.

## Additional information

No additional information is available for this paper.

## Funding

None to declare.

## Ethics approval

The research ethics were approved by the Can Tho University of Medicine and Pharmacy, Vietnam, code 22.148.SV/PCT-HDDD.

## Declaration of competing interest

The authors declare that they have no known competing financial interests or personal relationships that could have appeared to influence the work reported in this paper.
